# Are sperm parameters able to predict the success of assisted reproductive technology? A retrospective analysis of over 22,000 assisted reproductive technology cycles

**DOI:** 10.1111/andr.13123

**Published:** 2021-11-12

**Authors:** Maria Teresa Villani, Daria Morini, Giorgia Spaggiari, Angela Immacolata Falbo, Beatrice Melli, Giovanni Battista La Sala, Marilina Romeo, Manuela Simoni, Lorenzo Aguzzoli, Daniele Santi

**Affiliations:** ^1^ Department of Obstetrics and Gynaecology Fertility Centre Azienda Unità Sanitaria Locale‐IRCCS di Reggio Emilia Reggio Emilia Italy; ^2^ Department of Medical Specialties Unit of Endocrinology Ospedale Civile of Baggiovara Azienda Ospedaliero‐Universitaria of Modena Modena Italy; ^3^ Clinical and Experimental Medicine PhD Program University of Modena and Reggio Emilia Modena Italy; ^4^ Department of Biomedical, Metabolic and Neural Sciences Unit of Endocrinology University of Modena and Reggio Emilia Modena Italy

**Keywords:** assisted reproduction, fertilization rate, male infertility, sperm morphology, sperm motility

## Abstract

**Background:**

An explosive increase in couples attending assisted reproductive technology has been recently observed, despite an overall success rate of about 20%–30%. Considering the assisted reproductive technology‐related economic and psycho‐social costs, the improvement of these percentages is extremely relevant. However, in the identification of predictive markers of assisted reproductive technology success, male parameters are largely underestimated so far.

**Study design:**

Retrospective, observational study.

**Objectives:**

To evaluate whether conventional semen parameters could predict assisted reproductive technology success.

**Materials and methods:**

All couples attending a single third‐level fertility center from 1992 to 2020 were retrospectively enrolled, collecting all semen and assisted reproductive technology parameters of fresh cycles. Fertilization rate was the primary end‐point, representing a parameter immediately dependent on male contribution. Pregnancy and live birth rates were considered in relation to semen variables. Statistical analyses were performed using the parameters obtained according to the World Health Organization manual editions used for semen analysis.

**Results:**

Note that, 22,013 in vitro fertilization and intracytoplasmic sperm injection cycles were considered. Overall, fertilization rate was significantly lower in patients with abnormal semen parameters compared to normozoospermic men, irrespective of the World Health Organization manual edition. In the in vitro fertilization setting, both progressive motility (*p* = 0.012) and motility after capacitation (*p* = 0.002) significantly predicted the fertilization rate (statistical accuracy = 71.1%). Sperm motilities also predicted pregnancy (*p* < 0.001) and live birth (*p* = 0.001) rates. In intracytoplasmic sperm injection cycles, sperm morphology predicted fertilization rate (*p* = 0.001, statistical accuracy = 90.3%). Sperm morphology significantly predicted both pregnancy (*p* < 0.001) and live birth (*p* < 0.001) rates and a cut‐off of 5.5% was identified as a threshold to predict clinical pregnancy (area under the curve = 0.811, *p* < 0.001).

**Discussion:**

Interestingly, sperm motility plays a role in predicting in vitro fertilization success, while sperm morphology is the relevant parameter in intracytoplasmic sperm injection cycles. These parameters may be considered reliable tools to measure the male role on ART outcomes, potentially impacting the clinical management of infertile couples.

## 1 INTRODUCTION

The advent of assisted reproductive technology (ART) represented one of the most relevant medical innovations of the last century, drastically changing reproductive medicine. Different techniques and several treatment protocols evolved since the ART debut in 1978, but the vast majority of research studies in this setting remain focused on the same question, that is, how to increase ART success. To this aim, the identification of reliable and accurate predictors of ART outcome is mandatory. Since the use of assisted fertilization is accompanied by considerable economic and psycho‐social costs,^1^ the detection of predictive markers would be very useful to choose the ART type. However, the vast majority of studies evaluated only the female partner, whereas the male counterpart was relegated to a secondary role.

The studies considering common sperm parameters obtained by conventional semen analysis, that is, sperm number, motility, and morphology,^2^ generated important threshold values that are commonly applied in clinical practice. Considering the less artificial ART approach, that is, intrauterine insemination (IUI), the total number of progressively motile sperm detected in fresh ejaculate resulted not predictive in terms of pregnancy achievement.^3^, ^4^ However, the number of inseminated, progressively motile spermatozoa was detected as a reliable predictor of pregnancy,^5^ using one million as the clinical decision threshold to discriminate whether to send the couple to in vitro fertilization (IVF) or to IUI procedures.^4^, ^6^ However, infertile couples are more often addressed to more invasive techniques, such as IVF, irrespective of sperm quality‐related parameters. In the IVF setting, in which the embryo is obtained placing the spermatozoa and the oocyte in the same culture medium,^7^ the dependency of the outcome from sperm motility is expected.^8^ Accordingly, sperm motility thresholds are recommended to opt for IVF, that is, total sperm motility higher than 30% and progressive motility higher than 15%.^9^ Although it is reasonable to assume that sperm kinematic parameters could be relevant to improve IVF success rate, the current literature shows conflicting results, reporting a direct correlation between sperm progressive motility and pregnancy rate in some cohorts,^8^, ^10^ but not in others.^11^ Another sperm quality‐related parameter investigated in this setting is sperm morphology. However, the probability of IVF fertilization success seems to be independent of the percentage of normal forms, excluding its role as a reliable prognostic marker.^12^ Since the advent of the intracytoplasmic sperm injection (ICSI), the use of conventional IVF progressively decreased.^13^ Despite the direct microinjection of a single spermatozoon into the oocyte, the use of non‐motile spermatozoa seemed to negatively impact fertilization outcome.^5^ In addition, similarly to IVF, the prognostic value of sperm morphology remains poor also in the ICSI context.^12^ Indeed, a meta‐analytic approach failed to detect any association between isolated teratozoospermia and pregnancy rate, independently from the ART procedure used.^14^ The potential contribution of the male gamete to the final ART outcome has been evaluated using innovative indicators of sperm quality, such as sperm DNA fragmentation index (DFI).^15^ It has been hypothesized that an increased sperm DNA damage, exceeding 15%, could induce apoptotic pathways’ activation, promoting embryo arrest.^16^, ^17^ However, conflicting results emerged even about DFI, since meta‐analyses reported both a reduction^18^ and no difference^19^ in pregnancy rates in cases of high DFI levels in IVF and ICSI cycles.

Obviously, no convincing evidence that ART outcomes may be dependent on sperm parameters exists. Moreover, the variation over the years of the reference values in semen analysis, together with the poor inter‐laboratory standardization of sperm assessment, contributes to this very complex scenario.^20^ It remains undeniable that identifying thresholds of seminal parameters with prognostic significance in terms of pregnancy rate would have a considerable clinical impact in the assisted reproduction field. In this study, we aimed to answer the question of whether and which semen parameters can be useful for a priori prediction of ART outcomes. For this purpose, a large retrospective cohort analysis was conducted in a single third‐level fertility center.

## 2 MATERIALS AND METHODS

### 2.1 Study design and participants

A single‐center, retrospective, observational study was carried out. All couples attending the Santa Maria Nuova Hospital of Reggio Emilia (Reggio Emilia, Italy) for primary or secondary infertility between January 1992 and December 2020 were considered eligible. During the time‐frame interval evaluated, two historical moments (2005–2008 and 2018–2020) should be carefully considered, since structural interventions in the center and the COVID‐19 pandemic reduced the number of fresh ART cycles performed.

Couple infertility was defined as the absence of conception after 12 months of unprotected sexual intercourse.^21^, ^22^ Both partners were aged over 18 years and satisfied national criteria to access ART procedures. Only fresh ART cycles performed were considered for this study and each couple could be enrolled more than once, if undergoing more than one fresh cycle.

The embryo production and transfer were regulated by specific national laws. From 1992 to 2004, all oocytes retrieved were inseminated and a maximum of five embryos were transferred. Since 2004, law number 40 (L40/2004) established the maximum number of inseminated oocytes and embryos transferred to be three. In June 2009, the law was amended and, until March 2016, the maximum number of oocytes to be inseminated was set as follows: i) four for women younger than 38 years, ii) five for women aged between 38 and 39 years and iii) all available for women older than 40 years. Similarly, the maximum number of transferred embryos was two for women younger than 38 years, three for women aged between 38 and 39 years, and five for women older than 40 years. All embryos, formed but not transferred, could be frozen only since 2004. Between April 2016 and June 2020, all oocytes retrieved could be inseminated for all women independent of age. The number of maximum oocytes transferred was set to two for women younger than 38 years, to three for women aged between 38 and 39 years, and to four for women older than 40 years. Until July 2020, all embryos were transferred at the cleavage stage. Finally, in July 2020, only the number of embryos transferred was regulated. Accordingly, the transfer was performed at the blastocyst stage, with a maximum number allowed of one embryo for women younger and two for women older than 38 years.

### 2.2 ART procedures

Down‐regulation was obtained by gonadotropin‐releasing hormone agonists (GnRHa) (Enantone, Takeda Pharmaceutical, or Decapeptyl, Ipsen) applying a mild stimulation with the GnRHa protocol. Ovarian stimulation was performed applying different protocols: i) recombinant follicle‐stimulating hormone (FSH) alone (Gonal‐F, Merck Serono), ii) recombinant FSH plus luteinizing hormone (LH) (Pergoveris, Merck Serono), iii) highly purified human menopausal gonadotropin (Meropur, Ferring), or iv) biosimilar FSH (Ovaleap, Theramex Italy). Gonadotropin administration was started when serum estradiol levels were below 50 pg/ml and no ovarian follicles higher than 10 mm were detected. Ovarian stimulation was monitored by serum estradiol concentrations and serial ultrasonography evaluations. When more than three follicles with a diameter higher than 17 mm were observed at ultrasonography, human chorionic gonadotropin (hCG) (Gonasi, IBSA Institut Biochimique) was injected to complete oocyte maturation. Oocyte retrieval was performed 34–36 h after hCG administration by ultrasound‐guided transvaginal aspiration. All patients received supplemental progesterone for 15 days until β‐hCG assay.

Oocytes selection was performed selecting oocytes in metaphase II considered for ICSI. In IVF, the evaluation of nuclear maturity occurs indirectly through the morphological classification of the cumulus‐oocyte complexes (COC) during the oocyte retrieval. Only the mature and post‐mature COCs are subjected to IVF, even if in the absence of these, the immature COCs but not the degenerate ones are subjected to IVF (the morphological evaluation of the COCs is attributable to four classifications: immature, mature, post‐mature, and degenerate).

For conventional IVF, oocytes were individually cultured in micro drops of the fresh medium under mineral oil with 100,000 activated spermatozoa. For ICSI, after the removal of the cumulus and corona cells, nuclear maturation assessment of oocytes was performed using an inverted microscope to ensure sperm injection in metaphase‐II oocytes only.

Semen sample preparation was performed as described elsewhere.^23^, ^24^ Briefly, an appropriate aliquot of fresh semen was diluted with 10 ml of buffered medium. After centrifugation (10 min at 800 x g at room temperature), the supernatant was removed and replaced by 5 ml of buffered medium. After a second centrifugation, the supernatant was removed once again, and the pellet was overlaid with 1 ml of medium and incubated (37°C, 6% CO_2_ in air) to separate motile spermatozoa by swim‐up. After liquefaction, the sample was concentrated by one centrifugation (1500 x g), and the pellet was removed in 1 ml of medium. At the end of the sperm separation technique chosen for the processing of the seminal sample intended for the insemination of the oocytes (e.g. swim‐up). At the operative level, the supernatant was eliminated and the sperm pellet obtained was resuspended or stratified in 0.5–1.0 ml in the culture medium. In this way, a fraction of capacitated spermatozoa was obtained, or motile spermatozoa capable of carrying out the acrosome reaction with the oocytes.

Oocyte fertilization was assessed at 18–20 h (day 1) from insemination/injection and confirmed by the presence of two pronuclei and the alignment of nucleolar precursor bodies. In all cases, embryonic development was assessed on days 2 and 3 (i.e., after 41–43 and 65–67 h from insemination/injection, respectively). The best‐quality embryos were transferred on day 2 or 3 after IVF/ICSI procedures, until July 2020, when blastocyst transfer was started.

The ART approach is routinely suggested by the clinician and established on the day of pick up. This decision is based on semen parameters. In particular, IVF was selected in case of (i) normozoospermia and (ii) semen parameters’ alterations with the detection of more than 1.5 million/ml of capacitated spermatozoa. On the contrary, ICSI was selected in case of (i) frozen cycles, (ii) semen parameters’ alterations with the detection of less than 1.5 million/ml of capacitated spermatozoa, (iii) previous ART failure, and (iv) immunological reason of infertility. These historical and strict criteria did not change over the time of observations.

Biochemical pregnancies were assessed 12 days after embryo transfer by a positive quantitative serum β‐hCG value higher than 10 IU/L. In the case of a positive biochemical pregnancy test, micronized progesterone support (Prometrium, Rottapharm Madaus, 200 mg twice daily or Crinone, Merck Serono) was continued until 35 days after embryo transfer.

### 2.3 Outcomes

The main outcome was fertilization rate, considered as the ratio between the number of fertilized oocytes and the number of injected/inseminated oocytes. This parameter was selected as the primary end‐point since it represents the earliest outcome in which the male contribution can be assessed. Thus, this parameter was selected to verify the male role in ART processes, using earlier parameters, compared to strong ART outcomes. Secondary endpoints were the strongest ART outcomes, that is, pregnancy and live birth rates. The pregnancy rate was considered both as biochemical and clinical pregnancy. The former was defined by the detection of high levels of hCG in serum or urine, the latter was diagnosed by ultrasonography visualization or clinical documentation of at least one fetus with a heartbeat (including ectopic pregnancies) expressed for 100 initiated cycles, aspiration cycles, or embryo transfer cycles. Moreover, semen parameters were collected as specified in the following paragraph.

### 2.4 Semen analysis

Semen analyses were performed according to the World Health Organization (WHO) manual available at the time of the ART cycle. All semen analyses were performed by the same laboratory, undergoing regular internal and external quality controls over the years, and awarded the European Society of Human Reproduction and Embryology quality certificate.^25^

Semen parameters were analyzed for volume, sperm count, sperm motility, and sperm morphology. Considering the wide time‐frame interval of our study, three versions of the WHO manual were consecutively used during the data collection.^26^, ^27^, ^28^ First, semen analyses have been performed using the 3rd edition,^26^ whereas in 1999, the 4th edition^27^ and in 2010, the 5th edition^28^ were introduced. Each WHO manual edition introduced methodological differences and changed reference ranges. Since the 1992 and 1999 WHO manuals showed only minor changes in both methodologies and reference ranges, the results obtained using the two editions were considered together. In particular, oligozoospermia was defined by sperm concentration <20 million/ml and/or total sperm count <40 million, asthenozoospermia by progressive motility <25% and/or total motility < 50%, and teratozoospermia by sperm morphology <30% (3rd edition)^26^ or 14% (4th edition).^27^ On the contrary, the 5th edition defined oligozoospermia for sperm concentration <15 million/ml and/or total sperm count <39 million, asthenozoospermia for progressive motility <32% and/or total motility <40%, and teratozoospermia for sperm morphology <4%.^28^

The capacitation test was routinely performed for all patients in each semen sample in order to select the proper ART methodologies to be applied. Capacitated spermatozoa were obtained by first selecting motile spermatozoa by a swim‐up technique and then incubating the motile gametes in the Biggers‐Whitten‐Whittingham medium containing 35 mg/ml human serum albumin for 6 h.^29^, ^30^, ^31^ The swim‐up technique used for capacitation was the same preparation method as was used to prepare spermatozoa.

### 2.5 Statistical analysis

The entire cohort was first analyzed by descriptive analyses, considering both the female and the male partners. Semen analysis parameters were considered according to the WHO manual used for the interpretation and the sperm alterations classification was compared considering the cut‐off suggested by the WHO manual used, as reported above.

A descriptive analysis of strong ART outcomes, that is, pregnancy and live birth rates, was provided for the entire cohort of cycles. Categorical variables were compared between ART techniques applied using Fisher exact test.

In order to evaluate the male role in ART, the fertilization rate was calculated as the ratio between the number of fertilized oocytes and the number of injected/inseminated mature oocytes. In particular, the fertilization rate in ICSI cycles considered the number of oocytes injected, whereas in IVF cycles the number of inseminated oocytes. The fertilization rate calculated was adjusted for the maximum number of embryos that could be fertilized and transferred on the basis of the law in force year by year, as reported above.

Considering only cycles with oocytes retrieved, fertilization rate was first compared between IVF and ICSI cycles, performing Mann‐Whitney U‐test. The fertilization rate was compared among categories created on semen parameters alterations, performing Kruskal‐Wallis test and applying Dunnet post hoc analyses. These analyses were performed three times. First, the unadjusted dataset was used. Second, the fertilization rate was divided by the number of ART cycles performed for each couple. Third, the fertilization rate obtained in each couple was divided by the number of embryos that could be transferred according to the national rules in force in the year in which the ART was performed.

In order to evaluate the role of semen parameters on fertilization rate, a correlation analysis was performed using the Spearman Rho test considering semen analysis as independent and fertilization rate as a dependent variable. Then, multivariate linear regression analysis was performed, using fertilization rate as the dependent variable and semen parameters, anthropometrical variables, and smoking habits as independent variables. The number of ART cycles performed for each enrolled couple was used to adjust the independent variable, as reported above. Since independent variables in our analysis could be correlated, we performed the variance inflation factor (VIF), in order to identify the correlation between independent variables and the strength of that correlation. VIFs ranged from 1 to infinity. A VIF = 1 suggested the lack of collinearity, VIFs between 1 and 5 suggested that there is a moderate correlation, and VIFs greater than 5 represented critical levels of multicollinearity.

Semen parameters were finally considered as predictive variables of the main ART outcome (i.e., pregnancy and live birth rates). Logistic regression analyses were performed setting the ART outcome as dependent variable and semen volume, sperm concentration, total sperm number, and sperm morphology as covariates. Among confounding factors, the number of ART cycles performed was included in the analysis. Finally, in order to identify potential cut‐offs, receiving operating characteristic (ROC) analyses were performed using pregnancy and live birth rates as state variables and predictive semen parameters as test variables.

Statistical analysis was performed using the Statistical Package for the Social Sciences software for Windows (version 26.0; SPSS Inc., Chicago, IL, USA). For all comparisons, *p* < 0.05 was considered statistically significant.

## 3 RESULTS

Twenty‐two thousand and thirteen fresh ART cycles were performed between January 1992 and December 2020. Among these, 1545 (7.0%) cycles involved couples with secondary infertility (Table 1). Considering the entire ART cohort, 5819 cycles were IVF (26.4%) and 16,194 were ICSI (73.6%).

**TABLE 1:** Couples’ characteristics. Data are reported as median (95% confidence interval). [CFTR = cystic fibrosis transmembrane conductance regulator]

	**Overall cohort**
Number of female partners	22,013
Female age (years)	36.4 (28.0–42.0)
Female weight (kg)	70.0 (56.0–103.0)
Female body mass index (kg/m^2^)	25.3 (20.5–32.7)
Number of male partners	22,013
Male age (years)	38.9 (30.1–48.8)
Male weight (kg)	61.0 (59.2–90.0)
Male body mass index (kg/m^2^)	22.6 (18.4–32.0)
Infertility causes	
Pelvic, *n* (%)	784 (3.6%)
Endometriosis, *n* (%)	1232 (5.6%)
Tubal, *n* (%)	2133 (9.7%)
Immunological, *n* (%)	15 (0.1%)
Idiopathic, *n* (%)	5943 (27%)
Advanced maternal age, *n* (%)	4014 (18.2%)
Previous pregnancies	
1, *n* (%)	3206 (14.6%)
2, *n* (%)	1295 (5.9%)
3, *n* (%)	475 (2.2%)
4, *n* (%)	199 (0.9%)
>5, *n* (%)	92 (0.4%)
Previous miscarriages	
1, *n* (%)	2859 (13.0%)
2, *n* (%)	1019 (4.6%)
3, *n* (%)	316 (1.4%)
4, *n* (%)	123 (0.6%)
>5, *n* (%)	51 (0.3%)
Previous pre‐term delivery	
1, *n* (%)	139 (0.6%)
2, *n* (%)	13 (0.1%)
Previous delivery	
1, *n* (%)	1386 (6.3%)
2, *n* (%)	108 (0.5%)
3, *n* (%)	29 (0.1%)
4, *n* (%)	16 (0.1%)
>5, *n* (%)	6 (0.1%)
Female abnormal karyotype, *n* (%)	49 (0.2%)
Female CFTR heterozygous mutations, *n* (%)	143 (0.6%)
Male abnormal karyotype, *n* (%)	165 (0.7%)
Male CFTR heterozygous mutations, *n* (%)	219 (1.0%)
Male Y‐chromosome microdeletions, *n*(%)	16 (0.1%)
Smoking habit	
Female, *n* (%)	1805 (8.2%)
Male, *n* (%)	2322 (10.5%)

Table 2 reports descriptive analyses of the male partner, dividing patients according to the WHO manual's version used for semen analysis, as reported in the methods section. Using WHO 5th edition manual, higher semen volume (*p* < 0.001) and total sperm number (*p* < 0.001) were detected compared to previous editions, together with lower sperm concentration (*p* = 0.004), progressive motility (*p* < 0.001), and sperm morphology (*p* < 0.001) (Table 3). The detection of higher semen volume could be explained by the higher accuracy of the method suggested in the WHO manual's 5th edition. Indeed, previous methodologies are well known to underestimate semen volume of 0.3–0.9 ml.^32^, ^33^

**TABLE 2:** Semen parameters of male partners classified according to the World Health Organisation manual. Data are reported as median (95% confidence interval). The *p*‐value was calculated by Mann‐Whitney U‐test. [OAT = oligo‐astheno‐teratozoospermia; WHO = World Health Organisation]

	**WHO previous editions**	**WHO V edition**	
	**(*n* = 13,112)**	**(*n* = 8901)**	***p*‐value**
Semen volume (ml)	1.5 (0.5–4.2)	2.7 (0.9–6.0)	<0.001
Sperm concentration (million/ml)	28.0 (0.3–110.0)	25.0 (0.2–102.4)	0.004
Total sperm number (million)	46.5 (0.4–210.0)	60.0 (0.4–336.5)	<0.001
Progressive sperm motility (%)	30.0 (0.5–63.8)	29.0 (0.5–55.0)	<0.001
Normal sperm morphology (%)	6.0 (0.0–33.0)	3.0 (0.0–10.0)	<0.001
Semen alteration			
Oligozoospermia *n* (%)	5402 (41.2%)	3359 (37.7%)	<0.001
Asthenozoospermia *n* (%)	8969 (68.4%)	4709 (52.9%)	<0.001
Teratozoospermia *n* (%)	7917 (60.4%)	4983 (56.0%)	<0.001
OAT *n* (%)	4641 (35.4%)	2706 (30.4%)	<0.001

**TABLE 3:** Assisted reproductive technology (ART) outcomes considering the entire cohort and dividing according to the ART applied. Data are reported as median (95% confidence interval). [FSH = follicle‐stimulating hormone; ICSI = intracytoplasmic sperm injection; IVF = in vitro fertilization; LH = luteinizing hormone]

	**Entire cohort (*n* = 22,013)**	**ICSI cycles (*n* = 16,194)**	**IVF cycles (*n* = 5819)**
Follicles >15 mm of diameter	4.0 (1.0–10.0)	4.0 (1.0–10.0)	4.0 (1.0–10.0)
Oocytes retrieved	5.0 (1.0–13.0)	6.0 (1.0–14.0)	4.0 (1.0–11.0)
Mature oocytes	5.0 (1.0–12.0)	6.0 (1.0–13.0)	4.0 (1.0–11.0)
Oocytes injected (ICSI cycles)	5.0 (1.0–18.0)	4.0 (1.0–10.0)	–
Oocytes inseminated (IVF cycles)	4.0 (1.0–10.0)	–	4.0 (1.0–10.0)
Oocytes fertilized	2.0 (0.0–7.0)	2.0 (0.0–6.0)	3.0 (0.0–7.0)
Fertilization rate (%)	31.0 (5.0–90.0)	31.9 (5.0–90.0)	30.7 (5.0–90.0)
Total embryos	2.0 (0.0–7.0)	2.0 (0.0–7.0)	2.0 (0.0–7.0)
Transferred embryos	1.0 (0.0–4.0)	1.0 (0.0–40)	1.0 (0.0–5.0)
Frozen embryos	1.0 (0.0–6.0)	1.0 (0.0–5.0)	1.0 (0.0–6.0)
Gonadotropin stimulation choice			
Urinary FSH, *n* (%)	12,296 (55.9%)	10,642 (65.7%)	1654 (28.4%)
Recombinant FSH, *n (*%)	5082 (23.1%)	3123 (19.3%)	1959 (33.7%)
Human menopausal gonadotropin, *n* (%)	4430 (20.1%)	2368 (14.6%)	2062 (35.4%
FSH + LH, *n* (%)	201 (0.9%)	61 (0.4%)	140 (2.4%)
Stimulation duration (days)	13.0 (9.0–20.0)	13.0 (9.0–20.0)	13.0 (9.0–20.0)
Total gonadotropin dosages (IU)	2775.0 (1050.0–7200.0)	2625.0 (1000.0–7200.0)	3075.0 (1100.0–6900.0)

Interestingly, sperm motility after capacitation was directly related to sperm concentration (Rho = 0.515, *p* < 0.001), total sperm number (Rho = 0.425, *p* < 0.001), progressive motility (Rho = 0.537, *p* < 0.001), and sperm morphology (Rho = 0.503, *p* < 0.001) independently of the manual edition. The different normal ranges recommended by the WHO manual editions led to a different frequency of sperm alterations (Table 2). Indeed, according to the WHO 5th edition manual, a lower frequency of oligo‐, astheno‐, and teratozoospermia was diagnosed compared to the previous editions (Table 2).

Considering strong ART outcomes, the overall pregnancy rate was 20.4% (4368 cycles) for biochemical and 20.2% (4314 cycles) for clinical pregnancies, respectively. Among the latter, the overall live birth rate was 63.3%. Interestingly, both biochemical (20.8% vs. 19.1%, *p* < 0.001) and clinical (20.7% vs. 18.5%, *p* < 0.001) pregnancy rates were significantly higher in ICSI than IVF cycles. On the contrary, the live birth rate was not significantly different between ART methodologies (64.0% in ICSI vs. 60.9% in IVF, *p* = 0.074). Table 3 summarizes the main ART outcomes.

Only 610 cycles (2.8%) were interrupted since no oocytes were retrieved after gonadotropins stimulation. Considering only cycles with oocytes retrieved, the fertilization rate was significantly higher in ICSI (42.4 ± 39.7%) compared to IVF cycles (38.4 ± 49.2%) (*F* = 27.343, *p* < 0.001). Since the fertilization rate was significantly different between the two ART methodologies, the following analyses were performed for each technique separately.

### 3.1 IVF cycles

**FIGURE 1:**
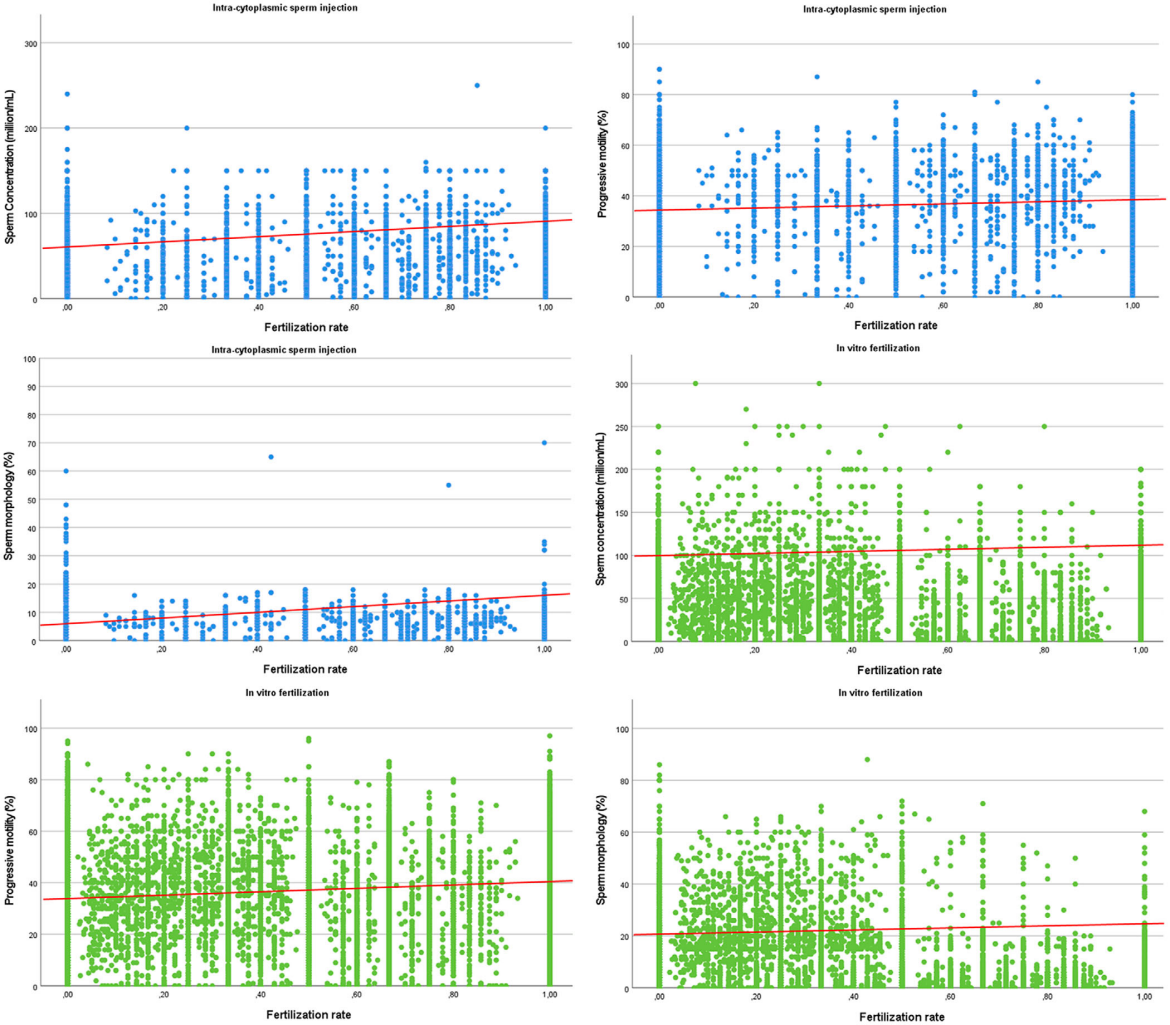
Box plots showing correlation between fertilization rate and sperm concentration (panel A and D), progressive motility (panel B and E), and sperm morphology (panel C and F). Redline shows the correlation trend

The fertilization rate was compared among classes of semen abnormalities, defined by the WHO manual's 5th edition and previous editions, separately (Table S1). Considering the WHO manual's 5th edition, fertilization rate was significantly lower in oligozoospermic patients (*p* = 0.030), in asthenozoospermic (*p* < 0.001), in teratozoospermic (*p* < 0.001), and in oligo‐astheno‐teratozoospermic (OAT) (*p* < 0.001), compared to normozoospermic patients (Table S1). Similar results were obtained using previous editions, showing lower fertilization rate in oligozoospermia (*p* < 0.001), asthenozoospermia (*p* < 0.001), teratozoospermia (*p* < 0.001), and OAT (*p* < 0.001), compared to normozoospermic patients (Table S1). As a confirmation, fertilization rate was directly correlated with sperm concentration (5th edition: Rho = 0.167, *p* < 0.001; previous editions: Rho = 0.346, *p* < 0.001), total sperm number (5th edition: Rho = 0.164, *p* < 0.001; previous editions: Rho = 0.342, *p* < 0.001), progressive motility (5th edition: Rho = 0.202, *p* < 0.001; previous editions: Rho = 0.274, *p* < 0.001) and sperm morphology (5th edition: Rho = 0.176, *p* < 0.001; previous editions: Rho = 0.599, *p* < 0.001), but not with semen volume (5th edition: Rho = 0.026, *p* = 0.138; previous editions: Rho = 0.048, *p* = 0.053) (Figure 1).

Multivariate analyses showed that only progressive motility according to the WHO manual's 5th edition was included in the final model (Table 4), predicting the overall fertilization rate. The multicollinearity evaluation showed a moderate correlation, not requiring further adjustment (VIF: total sperm count = 4.68, sperm concentration = 4.02, progressive sperm motility = 3.23, and sperm morphology = 2.14). Thus, the final statistical accuracy of the method was calculated as 71.1%.

**TABLE 4:** Multivariate linear regression analyses, divided for in vitro fertilization (IVF) and Intracytoplasmic sperm injection (ICSI) cycles

**In vitro fertilization (IVF)**			
	**Beta coefficient**	**95% confidence interval**	***p*‐value**
Semen volume (ml)	–0.150	0.717–1.035	0.110
Sperm concentration (million/ml)	–0.003	0.987–1.007	0.567
Total sperm number (million)	0.002	1.002–0.998	0.168
Progressive sperm motility (%)	0.175	1.020–1.120	<0.001
Normal sperm morphology (%)	–0.110	0.974–1.004	0.158
Male age (years)	0.020	0.980–1.062	0.325
Male body mass index (kg/m^2^)	–0.005	0.991–1.005	0.134
Male Smoking habit	0.030	1.004–0.967	0.267

**Intracytoplasmic sperm injection (ICSI)**			
Semen volume (ml)	0.030	1.004–0.987	0.110
Sperm concentration (million/ml)	–0.012	0.967–1.012	0.567
Total sperm number (million)	0.080	1.009–0.971	0.168
Progressive sperm motility (%)	0.020	0.980–1.062	0.168
Normal sperm morphology (%)	0.572	0.723–1.211	<0.001
Male age (years)	0.030	0.960–1.082	0.325
Male body mass index (kg/m^2^)	–0.010	0.990–1.009	0.134
Male Smoking habit	0.050	1.008–0.960	0.267

Considering strong ART outcomes, logistic regression analyses detected that sperm concentration (odds ratio [OR]: 1.007, 95% confidence interval [CI]: 1.005–1.009, *p* < 0.001), motility after capacitation (OR 1.009, 95% CI: 1.001–1.017, *p* < 0.001) and progressive motility (OR: 1.004, 95% CI: 1.001–1.006, *p* < 0.001) significantly predicted total pregnancy rate. On the contrary, the live birth rate was predicted only by sperm progressive motility (OR: 1.005, 95% CI: 1.001–1.007, *p* = 0.001). Since sperm motilities predicted pregnancy rate, ROC analyses were applied to detect possible sperm motility thresholds. However, neither progressive motility (area under the curve (AUC) = 0.483, standard error = 0.010, *p* = 0.102) nor motility after capacitation (AUC = 0.485, standard error = 0.018, *p* = 0.404) detected significant cut‐offs that could be exploited in clinical practice. Table S2 shows the average sperm motility and morphology dividing couples who achieved and who did not achieve pregnancy.

### 3.2 ICSI cycles

The fertilization rate after ICSI was significantly lower in oligozoospermic (*p* < 0.001), asthenozoospermic (*p* < 0.001), teratozoospermic (*p* = 0.003), and OAT (*p* < 0.001), compared to normozoospermic patients using the WHO manual's 5th edition (Table S1). This difference was confirmed using parameters obtained according to previous WHO manual editions, with lower fertilization rate in oligozoospermia (*p* < 0.001), asthenozoospermia (*p* < 0.001), teratozoospermia (*p* < 0.001), and OAT patients (*p* < 0.001), compared to normozoospermic subjects (Table S1). As detected in IVF, ICSI‐derived fertilization rate was directly correlated with sperm concentration (5th edition: Rho = 0.078, *p* < 0.001; previous edition: Rho = 0.264, *p* < 0.001), total sperm count (5th edition: Rho = 0.082, *p* < 0.001; previous edition: Rho = 0.239, *p* < 0.001), progressive motility (5th edition: Rho = 0.118, *p* < 0.001; previous edition: Rho = 0.277, *p* < 0.001) and sperm morphology (5th edition: Rho = 0.0.72, *p* < 0.001; previous edition: Rho = 0.237, *p* < 0.001), but not with semen volume (5th edition: Rho = 0.022, *p* = 0.117; previous edition: Rho = –0.038, *p* = 0.072), independently from the manual edition (Figure 1).

Multivariate analyses showed that only sperm morphology was included in the final model, predicting the overall fertilization rate (Table 4). The multicollinearity evaluation showed a moderate correlation, not requiring further adjustment (VIF: total sperm count = 4.91, sperm concentration = 4.76, progressive sperm motility = 4.21, and sperm morphology = 1.89). The final test accuracy was calculated at 90.3%.

**FIGURE 2:**
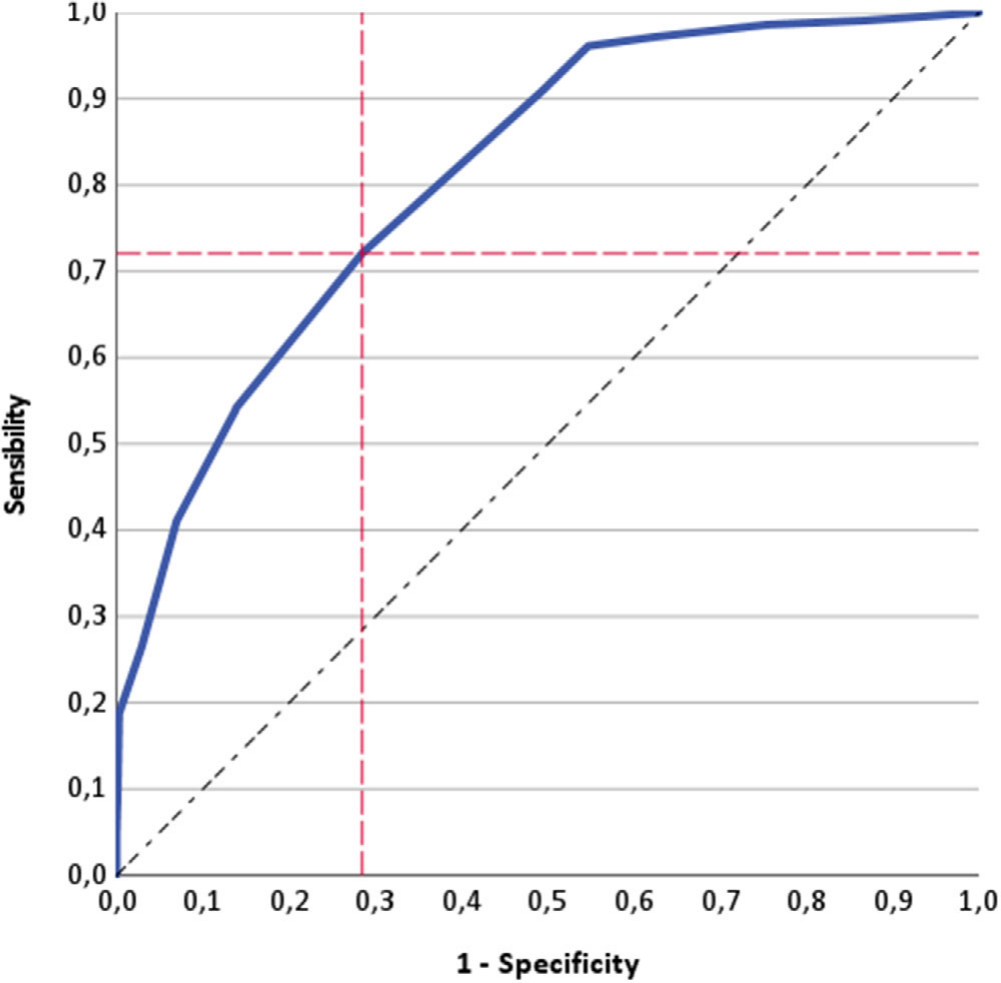
Receiving operating characteristic (ROC) analysis using clinical pregnancy rate as a state variable and sperm morphology as test variable in intracytoplasmic sperm injection cycles

Finally, applying logistic regression analyses to strong ART outcomes prediction, sperm morphology significantly predicted both pregnancy (OR: 1.020, 95% CI: 1.008–1.032, *p* < 0.001) and live birth rates (OR: 1.018, 95% CI: 1.013–1.023, *p* < 0.001). These results confirm the potential predictive role of sperm morphology for the final ICSI outcome. Therefore, we performed ROC analyses using sperm morphology as a test variable and pregnancy and live birth rates as independent state variables. The first ROC analysis showed that a sperm morphology cut‐off of 5.5% could be selected to predict clinical pregnancy (AUC = 0.811, standard error = 0.009, *p* < 0.001) with a sensitivity of 72% and specificity of 71% (Figure 2).

On the contrary, the ROC analysis performed using live birth rate as a state variable was not able to detect a sperm morphology threshold (AUC = 0.514, standard error = 0.024, *p* = 0.550). Table S2 shows the average sperm motility and morphology dividing couples who achieved and who did not achieve pregnancy.

## 4 DISCUSSION

In our large cohort of fresh cycles, we confirm a higher fertilization rate in ICSI compared to IVF, as demonstrated in a recent meta‐analysis.^34^ This parameter could be considered the earliest and most suited variable to evaluate the male contribution to oocyte activation.^35^ Indeed, fertilization rate, defined as the ratio between fertilized oocytes and injected/inseminated oocytes, is an informative, early parameter of the interaction of sperm plasma membrane and acrosome region with the oocyte within the sperm‐cumulus oophorus complex, leading to egg activation.^36^, ^37^ Thus, the fertilization rate could represent the first and the most reliable tool to evaluate the male role in IVF and ICSI cycles. In our cohort, the fertilization rate reached the highest percentage when all semen parameters are above the lower limit of the reference ranges, irrespective of the ART technique applied and of the WHO manual's version used for semen analysis. Indeed, a direct correlation between fertilization rate and semen parameters (i.e., sperm count, motility, and morphology) is detected in both ICSI and IVF cycles. However, applying more complex statistical analyses including all semen parameters together, fertilization rate is related to sperm morphology in ICSI cycles and to sperm motility in IVF cycles. These predictors are extremely interesting, suggesting and confirming the difference between the two ART methods, as well as the different male contributions to the ART according to the technique applied. However, considering only IVF cycles, the sperm morphology predictive ability is confirmed only when the WHO manual's 5th edition is used for semen analysis. Indeed, using previous editions, several semen parameters seem to influence fertilization rate, but they disappear when co‐variates are considered. With this in mind, we could speculate that the last WHO manual is more stringent and accurate in the definition of normal male fertility.^38^ However, differences obtained in semen alteration abnormalities should be carefully evaluated. Indeed, it could be the result of both the change in reference ranges suggested by the manual and the shift in lab practice, applied to follow the guidelines reported by WHO for the execution of semen analysis.

While conventional IVF relies on co‐incubation of oocytes with spermatozoa, ICSI consists of the injection of a single spermatozoon into the oocyte cytoplasm.^39^ For this difference, ICSI was initially employed only for the treatment of infertility due to a severe male factor. However, nowadays it is largely preferred in clinical practice, up to 65% of the cycles, irrespective of semen quality.^40^, ^41^ This high and perhaps excessive use of ICSI is reflected by its routine use in many ART programs, and its preferential application in case of unexplained couple infertility and/or isolated low morphology on semen analysis.^42^ However, despite ICSI representing one of the greatest advances in the field of assisted reproduction, bestowing men with severe oligozoospermia the opportunity to produce genetically own offspring, it does not seem to confer a concrete advantage over IVF in non‐male‐factor infertility.^43^, ^44^ Indeed, several studies suggested a higher risk of chromosomal abnormalities,^45^ epigenetic modifications,^46^ imprinting disorders,^47^ autism,^48^ intellectual disability,^49^ hospitalization at neonatal intensive care units,^50^ and congenital disorders^51^ in ICSI as compared to IVF cycles. It should be remembered that some of these conditions seem to be paternal age‐related, such as schizophrenia,^52^ Down,^53^, ^54^ and Apert^53^ syndromes. Moreover, ICSI is more time‐ and resource‐consuming compared to IVF.^55^ As a confirmation, the ICSI cost‐effectiveness was evaluated by some authors showing that a 3.3% increase in live birth rate could be achieved with an incremental cost of $29,666 compared to IVF.^56^ Here, we confirm a greater ICSI application compared to IVF in a large series of over 20,000 fresh cycles. In our large cohort, we confirm the superiority of ICSI over IVF in terms of strong ART outcomes as previously demonstrated. Indeed, several uncontrolled studies suggested that the pregnancy rate ranged from 9% to 12% in IVF^57^ and from 16% to 26% in ICSI cycles.^58^, ^59^, ^60^ However, the real reason for this difference is not clear. Potentially, ICSI cycles were initially applied to couples with male infertility causes, in which normal female fertility could lead to better quality embryos with superior implantation rates.^61^ However, in our cohort, although ICSI results in higher fertilization and pregnancy rates compared to IVF, the final live birth rate appears to be comparable between the two techniques, suggesting that the initial difference, in terms of fertilization and pregnancy rates, is finally smoothened by pregnancy and delivery‐related variables.

In this study, we demonstrate that pregnancy and live birth rates in ICSI cycles are predicted by sperm morphology. The decisional classification analysis suggests a higher ICSI success rate when the sperm morphology is higher than 5.5%. Thus, we could conclude that ICSI should be reserved for infertile couples in whom sperm morphology is good or after treatment of the male partner aiming to improve sperm morphology. Thus, a hypothetical sperm morphology improvement (i.e., after gonadotropins stimulation) could lead to a significant fertilization rate increase. Indeed, the current application of ICSI is not justified by the minimal increase of live birth rate obtained at disproportionately increased costs. Our result confirms the previous demonstration of the relevant role of sperm morphology in ART outcomes.^62^, ^63^, ^64^ The impact of teratozoospermia on IVF/ICSI offspring was inconclusive so far.^35^ Recently, Zhou et al. evaluated the influence of teratozoospermia on pregnancy outcome and newborn status after IVF and ICSI, detecting a limited predictive value for pregnancy outcomes and little impact on the resulting offspring.^65^ However, these results were obtained in a much smaller cohort of 2202 IVF cycles and 2574 ICSI cycles^65^ compared to the present study. Thus, we could speculate that the role of sperm morphology in ART outcomes emerges only by observing very large case series (statistical power 90.3%). Moreover, sperm morphology could represent an indirect measure of the real sperm quality. Indeed, in a very recent retrospective study, the relationship between DFI, a direct measure of DNA quality, and sperm morphology was investigated.^66^ The DFI‐morphology correlation was observed only in the motile sperm population identified after the swim‐up performed to select the spermatozoon subsequently used in ICSI or IVF procedures.^66^ The authors concluded that in case of detection of DFI ≥ 15% in the whole semen sample, the DFI analysis should be performed in spermatozoa selected after pellet swim up, to avoid picking out a spermatozoon presenting a normal morphology but a fragmented DNA.^66^ This result suggests that, although a spermatozoon with an apparently normal morphology could exhibit high DFI, causing a reduced embryo quality and pregnancy rate after ICSI,^67^ these two parameters are strictly related, highlighting the role that sperm morphology, if well performed, can play in clinical practice. A well‐shaped (nice‐looking) sperm is probably a healthy sperm, influencing the final reproductive result. The different impact of sperm morphology on IVF and ICSI cycles, suggests that the embryologist work should be tailored to the ART methods applied, in order to improve sperm selection.

Different from ICSI, better IVF results, in terms of fertilization and pregnancy rates, are achieved in those couples in whom the male partner shows the highest sperm count and motility. Thus, we could speculate that IVF should be reserved for those cycles with normal sperm count and progressive motility, rather than sperm morphology. In this setting, similar results are obtained using motility after capacitation, rather than progressive motility alone. Thus, this parameter seems to be useful to help the clinician to select the appropriate ART cycle to apply in a given infertile couple.^68^ Indeed, sperm capacitation, resulting in hyperactivated motility and acrosome reaction, naturally occurs for fertilization.^69^ It is well known that despite normal semen parameters, some semen samples fail to fertilize the oocyte since the sperm is not able to capacitate.^69^ With this in mind, it is clear that the evaluation of sperm motility after capacitation before IVF could be essential to better select the patient candidate for this ART technique. However, the final reproductive outcome, that is childbirth, seems to be unaffected by sperm motility. This result suggests that many other factors interfere with IVF success, other than sperm parameters. Thus, changing the point of view, the use of live birth rate as the outcome to evaluate male fertility‐related treatment is incorrect and too far from male contribution.

Our results must be considered cautiously since the Italian law changing over time significantly impacted the efficacy of ART approaches. Indeed, the interpretation of the Constitutional Court of law number 40 in 2009 introduced important limits to ART, limiting the number of embryos transferred and contributing to increasing the use of cryopreserved oocytes. This change reduced ART efficacy in couples in which the woman's age was higher than 38 years and in couples in which the testicular sperm extraction was required.^70^, ^71^, ^72^ However, in our cohort, we evaluated only fresh cycles and the mean female age was below this threshold, reducing this potential bias on our results. However, this selection reduced the sample size, probably reducing the final overall pregnancy rate obtained in the center. Moreover, this result should be due also to the long period of data collection, including two historical moments (2005–2008 and 2018–2020), in which structural interventions in the center and the COVID‐19 pandemic reduced the number of fresh ART cycles performed. Moreover, our results, although statistically significant, show confidence intervals at the limits of significance. Therefore, this data must be carefully considered. Indeed, from entering the ART path to its outcome, the variables encountered are numerous. However, the identification of a single parameter that influences, albeit slightly, the final outcome, indicates a considerable clinical weight.

In conclusion, our results, derived from a large and homogenous cohort followed in a center, confirm the clinical relevance of semen parameters in the ART setting. In particular, sperm motility seems to be the best male parameter to predict fertilization rate in IVF. On the contrary, sperm morphology shows the strongest relationship in ICSI cycles, considering either fertilization rate or final ART outcomes, such as pregnancy rate. This result could have an immediate transposition into clinical practice, helping the choice of the best ART approach to be performed on the infertile couple or suggesting therapy for the male partner to pursue an improvement in sperm motility/morphology.

## FUNDING INFORMATION

This study did not receive funds.

## CONFLICT OF INTEREST

The authors declare that they have no conflict of interest.

## AUTHOR CONTRIBUTIONS

Study design: Maria Teresa Villani, Daria Morini, Giorgia Spaggiari, Manuela Simoni, Daniele Santi. Data acquisition: Maria Teresa Villani, Daria Morini, Giorgia Spaggiari, Angela Immacolata Falbo, Beatrice Melli, GGiovanni Battista La Sala, Lorenzo Aguzzoli, Daniele Santi. Data analysis: Giorgia Spaggiari, MR, Daniele Santi. Drafting the manuscript: Giorgia Spaggiari, MR, Daniele Santi. Revising the manuscript: Maria Teresa Villani, Daria Morini, Giorgia Spaggiari, Angela Immacolata Falbo, Beatrice Melli, Giovanni Battista La Sala, MR, Manuela Simoni, Lorenzo Aguzzoli, Daniele Santi. Approval of the final version: Maria Teresa Villani, Daria Morini, Giorgia Spaggiari, Angela Immacolata Falbo, Beatrice Melli, Giovanni Battista La Sala, MR, Manuela Simoni, Lorenzo Aguzzoli, Daniele Santi.

## Supporting information

Supporting Information
