# Supplementary material for: Are sperm parameters able to predict the success of assisted reproductive technology? A retrospective analysis of over 22,000 assisted reproductive technology cycles
Source: Andrology. 2021 Nov 12;10(2):310–21. doi: 10.1111/andr.13123 (PMC9298690; doi:10.1111/andr.13123)
Supplement: Supplementary file 1 — Supporting Information [file ANDR-10-310-s001.docx]

**Supplementary Table 1**. Semen parameters, assisted reproductive technology (ART) parameters and ART outcomes dividing the entire cohort according to sperm parameters alterations suggested by the World Health Organisation manual. Data are reported as median (95% confidence interval).

[Astheno= asthenozoospermia; ICSI= intracytoplasmic sperm injection; IVF= *in vitro* fertilization; Normo= normozoospermia; OAT= oligo-astheno-teratozoospermia; Oligo= oligozoospermia; Terato= teratozoospermia; WHO= World Health Organization]

|  | **WHO previous editions** | | | | | **WHO V edition** | | | | |
| --- | --- | --- | --- | --- | --- | --- | --- | --- | --- | --- |
|  | **Normo** | **Oligo** | **Astheno** | **Terato** | **OAT** | **Normo** | **Oligo** | **Astheno** | **Terato** | **OAT** |
| **Semen volume (mL)** | 1.5  (1.5 – 4.0) | 2.3 (1.5 – 4.5) | 1.5 (1.5 – 4.5) | 1.5 (1.5 – 4.0) | 1.5 (1.5 – 4.5) | 1.5 (1.5 – 4.0) | 2.8 (0.7 – 6.5) | 2.5 (0.6 – 6.0) | 2.5 (0.7 – 6.0) | 2.5 (0.5 – 6.0) |
| **Sperm concentration (million/mL)** | 48.0 (19.0 – 120.0) | 4.3 (0.1 – 17.0) | 17.0 (0.1 – 85.0) | 11.0 (0.1 – 61.0) | 3.7 (0.1 – 18.0) | 42.0 (15.0 – 120.0) | 4.0 (0.1 – 14.0) | 9.0 (0.1 – 70.0) | 10.0 (0.1 – 70.0) | 2.5 (0.1 – 13.0) |
| **Total sperm number (million)** | 81.0 (30.0 – 245.0) | 7.5 (0.1 – 38.0) | 30.0 (0.1 – 170.0) | 19.5 (0.1 – 120.0) | 6.0 (0.1 – 34.0) | 108.0 (40.0 – 385.0) | 8.3 (0.1 – 38) | 21 (0.2 – 220.0) | 23.2 (0.2 – 220.0) | 5.4 (0.1 – 38.0) |
| **Progressive sperm motility (%)** | 49.0 (40.0 –68.0) | 15.0 (0.1 – 48.0) | 21.0 (0.1 – 39.0) | 21.0 (0.1 – 55.0) | 11.0 (0.1 – 36.0) | 42.0 (32.0 –58.0) | 9.0 (0.1 – 40.0) | 13.0 (0.1 – 29.0) | 17.0 (0.1 – 48.0) | 2.0 (0.1 – 28.0) |
| **Sperm typical forms (%)** | 15.0 (14.0 – 40.0) | 2.0 (0.0 – 15.0) | 4.0 (0.0 – 26.0) | 3.0 (0.0 – 11.0) | 1.0 (0.0 – 8.0) | 6.0 (4.0 – 10.0) | 1.0 (0.0 – 6.0) | 1.0 (0.0 – 8.0) | 1.0 (0.0 – 4.0) | 1.0 (0.0 – 3.6) |
| **Follicles >15 mm of diameter (n)** | 3.0 (0.0 – 8.0) | 3.0 (0.0 – 8.0) | 3.0 (0.0 – 8.0) | 3.0 (0.0 – 8.0) | 4.0 (0.0 – 8.0) | 4.0 (1.0 – 10.0) | 4.0 (1.0 – 10.0) | 4.0 (1.0 – 10.0) | 4.0 (1.0 – 4.0) | 4.0 (1.0 – 10.0) |
| **Oocytes retrieved (n)** | 7.0 (1.0 – 13.0) | 6.0 (1.0 – 15.0) | 6.0 (1.0 – 14.0) | 6.0 (1.0 – 14.0) | 6.0 (1.0 – 14.0) | 7.0 (1.0 – 13.0) | 5.0 (1.0 – 11.0) | 5.0 (1.0 – 11.0) | 5.0 (1.0 – 11.0) | 5.0 (1.0 – 11.0) |
| **Mature oocytes (n)** | 7.0 (1.0 – 14.0) | 6.0 (1.0 – 15.0) | 6.0 (1.0 – 14.0) | 6.0 (1.0 – 13.0) | 6.0 (1.0 – 13.0) | 7.0 (1.0 – 1.0) | 5.0 (1.0 – 11.0) | 5.0 (1.0 – 11.0) | 5.0 (1.0 – 12.0) | 6.0 (1.0 – 13.0) |
| **Oocytes injected (ICSI) (n)** | 5.0 (0.0 – 10.0) | 4.0 (0.0 – 11.0) | 4.0 (1.0 – 10.0) | 4.0 (0.0 – 10.0) | 4.0 (0.0 – 11.0) | 5.0 (0.0 – 9.0) | 4.0 (0.0 – 10.0) | 4.0 (1.0 – 10.0) | 4.0 (0.0 – 10.0) | 3.0 (0.0 – 9.0) |
| **Oocytes inseminated (IVF) (n)** | 5.0 (1.0 – 10.0) | 4.0 (1.0 – 15.0) | 5.0 (1.0 – 20.0) | 5.0 (1.0 – 18.0) | 4.0 (1.0 – 13.0) | 5.0 (1.0 – 10.0) | 4.0 (1.0 – 10.0) | 5.0 (1.0 – 10.0) | 4.0 (1.0 – 10.0) | 4.0 (1.0 – 10.0) |
| **Oocytes fertilized (n)** | 3.0 (1.0 – 7.0) | 2.0 (0.0 – 5.0) | 2.0 (0.0 – 6.0) | 2.0 (0.0 – 6.0) | 2.0 (0.0 – 5.0) | 3.0 (1.0 – 7.0) | 2.0 (1.0 – 6.0) | 2.0 (1.0 – 6.0) | 2.0 (1.0 – 6.0) | 2.0 (0.0 – 6.0) |
| **Fertilization rate (%)** | 67.0 (0.5 – 80.0) | 65.0 (0.5 – 80.0) | 41.0 (0.5 – 80.0) | 47.0 (0.5 – 80.0) | 45.0 (0.5 – 90.0) | 66.7 (0.5 – 80.0) | 64.1 (0.5 – 90) | 40.0 (0.5 – 90.0) | 40.0 (0.5 – 80.0) | 43.0 (0.5 – 90.0) |
| **Total embryos (n)** | 2.0 (1.0 – 8.0) | 2.0 (0.0 – 6.0) | 2.0 (0.0 – 7.0) | 2.0 (0.0 - 7.0) | 2.0 (0.0 - 6.0) | 2.0 (1.0 – 8.0) | 2.0 (0.0 – 6.0) | 2.0 (0.0 – 760) | 2.0 (0.0 - 7.0) | 2.0 (0.0 - 6.0) |
| **Transferred embryos (n)** | 1.0 (0.0 – 4.0) | 1.0 (0.0 – 4.0) | 1.0 (0.0 – 4.0) | 1.0 (0.0 – 4.0) | 1.0 (0.0 – 4.0) | 1.0 (0.0 – 4.0) | 2.0 (0.0 – 4.0) | 2.0 (0.0 – 4.0) | 1.0 (0.0 – 4.0) | 2.0 (0.0 – 6.0) |
| **Frozen embryos (n)** | 1.0 (0.0 – 5.0) | 1.0 (0.0 – 3.0) | 1.0 (0.0 – 4.0) | 0.0 (0.0 – 4.0) | 0.0 (0.0 – 3.0) | 1.0 (0.0 – 5.0) | 2.0 (0.0 – 6.0) | 2.0 (0.0 – 6.0) | 0.0 (0.0 – 4.0) | 2.0 (0.0 – 4.0) |
| **Stimulation duration (days)** | 15.0 (10.0 – 16.0) | 15.0 (9.5 – 16.0) | 15.0 (10.0 – 15.0) | 15.0 (10.0 – 16.0) | 14.0 (10.0 – 16.0) | 13.0 (9.0 – 20.0) | 13.0 (9.0 – 20.0) | 13 (9.0 – 20.0) | 13.0 (9.0 – 20.0) | 13.0 (9.0 – 20.0) |
| **Total gonadotropin dosages (IU)** | 3375.0 (1200.0 – 9450.0) | 3375.0 (1280.0 – 9950.0) | 3500.0 (1350.0 – 9532.5) | 3300.0 (1200.0 – 9852.0) | 3375.0 (1367.5 – 9900.0) | 2700.0 (1050.0 – 6750.0) | 2475.0 (990.0 – 6300.0) | 2600.0 (1000.0 – 6300.0) | 2550.0 (1000.0 – 6375.0) | 2475.0 (975.5 – 6300.0) |
| **Biochemical pregnancy** | 1672 (20.5%) | 1068 (19.8%) | 1799 (20.1%) | 1751 (22.1%) | 923 (19.7%) | 1112 (19.5%) | 658 (19.6%) | 894 (19.0%) | 936 (18.8%) | 521 (19.3%) |
| **Clinic Pregnancy** | 1657 (20.4%) | 1058 (19.8%) | 1782 (19.9%) | 1734 (21.9%) | 915 (19.7%) | 1112 (19.1%) | 649 (19.3%) | 878 (18.7%) | 921 (18.5%) | 514 (19.0%) |

|  | **Couples who achieved pregnancy** | **Couples who not achieved pregnancy** |  |
| --- | --- | --- | --- |
| ***In vitro* fertilization** | | | |
| Sperm motility (%) | 6.4 + 9.9 | 8.8 + 5.8 |  |
| Sperm morphology (%) | 26.4 + 19.7 | 26.3 + 19.9 |  |
| **Intra-cytoplasmic sperm injection** | | | |
| Sperm motility (%) | 39.4 + 13.3 | 34.9 + 16.1 |  |
| Sperm morphology (%) | 8.8 + 5.8 | 6.5 + 6.1 |  |

**Supplementary table 2**. Sperm motility and sperm morphology in couples who achieved or who not achieved a pregnancy, divided according to the ART technique applied. Data are expressed as mean + standard deviation.
